# Plasma Metabolomics Reveals Pathogenesis of Retained Placenta in Dairy Cows

**DOI:** 10.3389/fvets.2021.697789

**Published:** 2021-08-11

**Authors:** Yuqiong Li, Zhengwei Zhao, Yang Yu, Xiaojun Liang, Shengyi Wang, Lei Wang, Dongan Cui, Meizhou Huang

**Affiliations:** ^1^Laboratory Institute of Animal Science, Ningxia Academy of Agricultural and Forestry Sciences, Yinchuan, China; ^2^Engineering and Technology Research Center of Traditional Chinese Veterinary Medicine of Gansu Province, Lanzhou Institute of Husbandry and Pharmaceutical Sciences of Chinese Academy of Agricultural Sciences, Lanzhou, China; ^3^Academician (Expert) Workstation of Sichuan Province, The Affiliated Hospital of Southwest Medical University, Luzhou, China

**Keywords:** retained placenta, dairy cow, metabolomics, biomarker, pathogenesis

## Abstract

The complex etiology and pathogenesis of retained placenta (RP) bring huge challenges for researchers and clinical veterinarians in investigating the pathogenesis and treatment schedule. This study aims to investigate the pathogenesis of RP in dairy cows by plasma metabolomics. As subjects, 10 dairy cows with RP and 10 healthy dairy cows were enrolled according to strict enrollment criteria. Imbalanced antioxidant capacity, reduced Th1/Th2 cytokine ratio, and deregulation of total bilirubin (T-bil), alkaline phosphatase (ALP), and reproductive hormones were shown in dairy cows with RP by detecting biochemical indicators, oxidation and antioxidant markers, and cytokines in serum. Plasma metabolites were detected and analyzed by a liquid chromatography–mass spectrometry (LC–MS) system coupled with multivariate statistical analysis software. A total of 23 potential biomarkers were uncovered in the plasma of dairy cows with RP. The metabolic pathways involved in these potential biomarkers are interconnected, and the conversion, utilization, and excretion of nitrogen were disturbed in dairy cows with RP. Moreover, these potential biomarkers are involved in the regulation of antioxidant capacity, inflammation, and autocrine or paracrine hormone. All of these findings suggest that an imbalance of these potential biomarkers might be responsible for the imbalanced antioxidant capacity, reduced Th1/Th2 cytokine ratio, and deregulation of reproductive hormones in dairy cows with RP. The regulation of metabolic pathways involved in these potential biomarkers represents a promising therapeutic strategy for RP.

## Introduction

The retained placenta (RP), a common multifactorial postpartum reproductive disease manifesting as failure to expel fetal membranes within 24 h of calving, increases the risk of developing metritis and infertility and reduces milk production and quality, causing great financial losses in the dairy industry (1–4). The etiology, pathogenesis, and therapy of RP have been extensively explored by many researchers (4–7). At present, the incidence of RP morbidity varies across countries and herds, which are closely related to the management and environment of herds; the physiological state of cows, namely, age, parity, heredity, hormones, and nutrition; and the condition of calves, such as stillbirth and twinning (4, 8). The complex etiology and pathogenesis bring huge challenges for researchers and clinical veterinarians in probing the pathogenesis and treatment schedule of RP (9–12). The pathogenesis of RP is still unclear, and there are four popular hypotheses to explain it: deregulation of uterine contractions, dysfunction of the chorionic villi, inflammatory stress, and immune disorders (13–15). Many studies have also confirmed that changes in blood metabolites, cytokines, inflammatory factors, immune factors, and hormones are associated with the pathogenesis of RP (3, 13, 16, 17). However, it is difficult to clarify the complex pathogenesis of RP involving the nutritional metabolic, immune, nervous, and reproductive systems through blood indicators.

Metabolomics, a fairly new branch of omics technologies, can monitor the overall changes of small-molecule metabolites under physiological or pathological conditions, which contributes to finding disease biomarkers, probing differential metabolic pathways in the development of disease, and clarifying the pathogenesis of disease (18–20). A large body of studies have confirmed that the biomarkers screened by metabolomics are not only involved in diagnosis but also implicated in exploring the pathogenesis of the disease (21–24). Changes in the profiles of metabolites can trace and predict the occurrence and development of disease, but they are also easily affected by the interference of multiple confounding factors. Therefore, it is essential for metabolomics to exclude confounding factors in the collection of disease samples.

A growing body of evidence supports the utilization of alterations in biochemical profiles, antioxidant ability, cytokines, and hormones as biological indicators of RP (3, 25, 26). It has also recognized that several reproductive hormones and Th1 and Th2 cells participate in the process of separation and discharge of the placenta (16, 26, 27). Besides, oxidative stress and alterations in biochemical profiles as reasons or results of RP have been extensively attended. In this study, several biochemical indicators, oxidation and antioxidant markers, and cytokines along with plasma metabolism were detected. Based on the differential metabolites and their targeted metabolic pathways, coupled with the changes in biochemical indicators, oxidation and antioxidant markers, and cytokines, targeted regulation of the metabolic state of dairy cows with RP may be beneficial to clarify the pathogenesis of the disease and develop an effective treatment schedule.

In the present study, to detect the pathogenesis of RP in dairy cows, untargeted plasma metabolism along with the detection of related biochemical indicators, oxidation and antioxidant markers, and cytokines were performed, which is helpful for clarifying the pathogenesis and developing an effective treatment schedule.

## Materials and Methods

### Chemicals and Materials

All LC solvents [methanol, acetonitrile (ACN), and isopropanol] were of liquid chromatography–mass spectrometry (LC–MS) grade and purchased from Fisher Scientific (Loughborough, UK). LC–MS additives (formic acid and ammonium acetate) were acquired from Sigma-Aldrich (Madrid, Spain). Lithocholic acid-D4 (LCAD4) was obtained from Steraloids (Steraloids Inc., Newport, RI, USA). Phenylalanine-D5 (Phe-D5) was purchased from Cambridge Isotope Laboratories (Tewksbury, MA, USA). An ACQUITY UPLC BEH amide column was purchased from Waters (Milford, MA, USA). A vacuum blood collection tube was purchased from Laiwu Yaohua Pharmaceutical Packing Co., Ltd. (Shandong, China). The commercial kits for blood biochemical parameters were provided by Ningbo Medical System Biotechnology Co., Ltd. (Ningbo, China). Malondialdehyde (code number: S0131S), Cu/Zn-SOD and Mn-SOD assay kit (code number: S0103), and Total Glutathione Peroxidase assay kit (code number: S0058) were purchased from Beyotime (Shanghai, China). Interleukin (IL)-2, IL-4, IL-10, and tumor necrosis factor alpha (TNF-α) enzyme-linked immunosorbent assay (ELISA) kits (code numbers: ab193682, ab277388, ab277386, and ab193683, respectively) were purchased from Abcam (Cambridge, USA).

### Animals and Clinical Specimens

In this study, the enrollment criteria of dairy cows with RP were drawn up to limit sample heterogeneity caused by confounding factors. All experimental procedures involving animals were conducted according to the guidelines of the Animal Care and Use Committee of the Lanzhou Institute of Husbandry and Pharmaceutical Sciences of Chinese Academy of Agricultural Sciences (Lanzhou, China) (Animal Use Permit: SCXK201808-1756). All animals were obtained from a dairy farm with 680 lactating Holstein–Friesian cows in northwestern China, and the annual incidence of RP was 10.4% at this dairy farm. In the present study, RP is defined as placenta that has not been expelled within 24 h postpartum. The dairy cows enrolled as healthy dairy cows were of the same parity as the dairy cows with RP and expelled placenta within 24 h postpartum. Besides, they were free of infectious or metabolic diseases. All enrolled dairy cows had a good mental and physical state and appetite, a body condition score (BCS) between 2.5 and 4.0 at the time of calving according to the criteria specified in the protocol of Edmonson et al. (28), and clinical history of veterinary quarantine and clinical records. The dairy cows with a history of cesarean section during previous or current calving, displaced abomasum, laminitis, or any drug therapy excluding preventive medication in the standardized management of dairy farms were excluded from the study. To limit the effect of confounding factors on metabolic profiles, according to the enrollment and exclusion criteria, 20 dairy cows were enrolled in the experiment: 10 healthy cows were set as the control group, and 10 cows with RP made up the disease group. The characteristics of enrolled dairy cows, namely, BCS at calving, parity, and age, are shown in Supplementary Table 1. The cows in both groups had similar physical characteristics. Because RP was defined as placenta that had not been expelled within 24 h postpartum, all blood samples were collected at 24 h after calving to explore metabolic alterations in dairy cows with RP. All enrolled dairy cows had a clinical history of veterinary quarantine and clinical records. Blood samples of enrolled cows were collected from the jugular vein at 24 ± 1 h postpartum in K2 EDTA anti-coagulation vacuum tubes or evacuated tubes without anticoagulant to obtain plasma and serum, respectively. In brief, the blood samples were left at room temperature for 1 h and then after centrifugation at 1,600 × *g* for 10 min at 4°C, the supernatants in the anti-coagulation vacuum tubes were transferred into sterile tubes without any preservatives and stored at −80°C until analysis. The clotted blood in the evacuated tubes without anticoagulant was centrifuged at 2,000 × *g* at 4°C for 20 min, and the supernatant was transferred into sterile tubes.

### Metabolite Sample Preparation

In the present study, the profiles of metabolites in plasma of dairy cows with RP were investigated by ultra-high liquid chromatography tandem mass spectrometry to screen the potential biomarkers and differential metabolic pathways of RP and explain its pathogenesis. For LC–MS metabolomics analysis, 100 μl of plasma was mixed with 400 μl of ice-cold ACN/methanol (1:1, v/v) for deproteinization. After vortexing (30 s), samples were allowed to rest at −20°C for 10 min and then centrifuged (14,000 × *g*, 10 min) at 4°C. The 400 μl supernatant of each sample was transferred to another tube and evaporated to dryness with a vacuum concentrator. Each dried sample was reconstituted in 40 μl of ACN/water (1:1, v/v), sonicated for 10 min, and then centrifuged for 15 min at 13,000 rpm. The supernatants were transferred to analytical vials and stored at −80°C prior to LC–MS analysis. We pooled plasma from all samples (100 μl) to create a single pooled quality control (QC) sample, which was prepared as described above.

### UPLC/QTOF-MS Analysis of Plasma Metabolites

Tables LC–MS/MS analyses were performed using an Agilent 1290 Infinity LC system (Agilent Technologies, Inc., Palo Alto, USA) coupled to a Triple TOF 5600 system (AB SCIEX, Foster City, CA, USA). Samples were analyzed through using an ACQUITY UPLC HSS T3 (1.8 μm, 2.1 × 100 mm columns; Waters) for HILIC analysis. Each sample was analyzed two times under positive and negative ionization modes. The temperature of the column and auto-sampler was maintained at 25 and 4°C, respectively. The injection volume was 2 μl, and the flow rate was 0.3 ml/min for both positive and negative ion modes. For the positive ionization mode, mobile phases A and B were 0.1% formic acid in deionized water and 0.1% formic acid in ACN. For the negative ionization mode, mobile phases A and B were 0.5 mmol ammonium fluoride in deionized water and ACN. The optimized gradient program is established as shown in Supplementary Table 2.

Mass spectrometry was operated by electrospray ionization in positive and negative ion modes. For the positive ion mode, collision energy was set to 50 V, declustering potential was set to 60 V, and ion spray voltage floating was set to +5,000 V; for the negative ion mode, collision energy was set to 20 V, declustering potential was set to −60 V, and ion spray voltage was set to −5,000 V. The other source parameters were as follows: ion source gas 1 was set at 40 psi, ion source gas 2 was set at 80 psi, curtain gas was set at 30 psi, and source temperature was set at 650°C. The data were acquired over a mass-to-charge ratio (m/z) range of 60–1,000 Da with a TOF MS scan rate of 0.20 s/spectrum and an m/z range of 25–1,000 for production ion scan with an accumulation time of 0.05 s/spectrum. The product ion scan was acquired by information dependent acquisition (IDA) with high sensitivity mode selected. The MS/MS conditions were set as follows: declustering potential: ±60 V; collision energy: 35 ± 15 eV; exclusion of isotopes: within 4 Da; and candidate ions to monitor per cycle: 6.

Plasma samples from the two groups were analyzed in random order during the analysis. In addition, QC samples were detected once every five subject samples for conditioning of the analytical system, signal correction, and quality assurance.

### Serum Biochemistry Analysis

The serum concentrations of total bilirubin (T-bil), total protein (TP), albumin (ALB), globulin (GLB), alanine aminotransferase (ALT), aspartate aminotransferase (AST), alkaline phosphatase (ALP), creatine kinase (CK), urea (BUN), creatinine (CREA), glucose (GLU), triglycerides (TG), and total cholesterol (TC) were detected by an automatic biochemical analyzer (ERBA XL 600®) according to the manufacturer's instructions for the corresponding commercial kits.

### Oxidation and Antioxidant Markers in Serum

The concentrations of malondialdehyde (MDA), superoxide dismutase (SOD), and glutathione peroxidase (GSH-Px) were probed through commercial kits according to the manufacturer's protocols. In brief, the serially diluted MDA standards and test serum samples were prepared and added to the microplate well, and then 10 μl of MDA color reagent stock solution was added into each well of MDA standard and serum sample and incubated at room temperature for 20 min. The 10 μl of reaction solution was added to each cell plate and incubated at room temperature for 60 min. The absorbance increase of each plate well was monitored by Enspire Microplate Reader at 695 nm.

### Th1/Th2 Cytokine Measurement

The serum levels of IL-2, IL-4, IL-10, and TNF-α were detected by the corresponding commercial ELISA kits according to the manufacturer's protocols. In brief, all reagents should be brought to room temperature before use. The serum samples or standard working solution samples were added to the corresponding ELISA microplate wells and combined with the specific antibody. Then, a biotinylated detection antibody and avidin–horseradish peroxidase (HRP) conjugate are added successively to each microplate well and incubated. Free components are washed away. The substrate solution is added to each well and incubated. The enzyme–substrate reaction is terminated by the addition of stop solution. The optical density (OD) is measured spectrophotometrically at a wavelength of 450 nm. The ratio of Th1 and Th2 was calculated according to the following formula: Th1/Th2 = (CIL-2 + CTNFα) / (CIL-4 + CIL10).

### Reproductive Hormone Analysis

The serum levels of estradiol, progesterone, and PGF2α in serum were detected by the Beijing North Institute of Biotechnology Co., Ltd. using the corresponding commercial assay kits. The detection protocol of estradiol, progesterone, and PGF2α was similar with that of TNF-α, as was already depicted in section Th1/Th2 Cytokine Measurement.

### Statistical Analysis

#### Multivariate Statistical Analysis of Plasma Metabolite Data

The raw MS data were processed by Progenesis QI (Nonlinear Dynamics, Newcastle, UK) to filter the noise, correct the baseline, align the peaks, and identify and quantify the peaks. Retention time errors of <0.1 min were applied to align the peaks. Ion peaks with missing values >50% in both groups were deleted from the alignment data. Then, the normalized data with auto-scaling were imported into MetaboAnalyst software online (29) to perform multivariate and single-dimensional statistical analysis, namely, unsupervised principal component analysis (PCA), supervised partial least squares discriminant analysis (PLS-DA), *t*-test, and fold change analysis. The potential biomarkers were selected in accordance with variable importance in projection (VIP) score >1 from the PLS-DA model. The potential biomarkers were further optimized by Student's *t*-test for their abundance in dairy cows with RP and healthy dairy cows. Adjusted *p*-value < 0.05 was considered to be statistically significant. The biomarkers were further screened in accordance with VIP score >1, adjusted *p*-value < 0.05, and fold change >2 or <0.5.

#### Statistical Analysis of BCS, Age, Serum Biochemistry, Oxidation and Antioxidant Markers, Th1/Th2 Cytokine, and Reproductive Hormone Data

The BCS, age, serum biochemistry, oxidation and antioxidant markers, Th1/Th2 cytokine, and reproductive hormone data were analyzed through SAS 9.2 (SAS Institute Inc., Cary, NC, USA). Differences of BCS, age, serum biochemistry, oxidation and antioxidant markers, Th1/Th2 cytokine, and reproductive hormone between healthy group and RP group were compared through one-way analysis of variance with Tukey's test.

### Biomarker Identification and Metabolic Pathway Analysis

Optimization of candidate biomarkers was performed by comparing the accuracy of the m/z values (<25 ppm), and the MS/MS spectra were interpreted using a self-built metabolite database (Shanghai Applied Protein Technology Co., Ltd., Shanghai, China) based on their MS and MS/MS signatures. In order to evaluate the rationality of candidate biomarkers and intuitively display the differences in expression patterns of candidate metabolites in different samples, hierarchical cluster analysis based on the abundance of candidate biomarkers was performed. Cluster and correlation analysis of the optimized candidate biomarkers was performed by R (version 3.6.1). The metabolic pathways involved in the optimized candidate biomarkers were identified by the Kyoto Encyclopedia of Genes and Genomes (KEGG) database.

## Results

### Metabolic Alterations in Dairy Cows With RP

**Figure 1:**
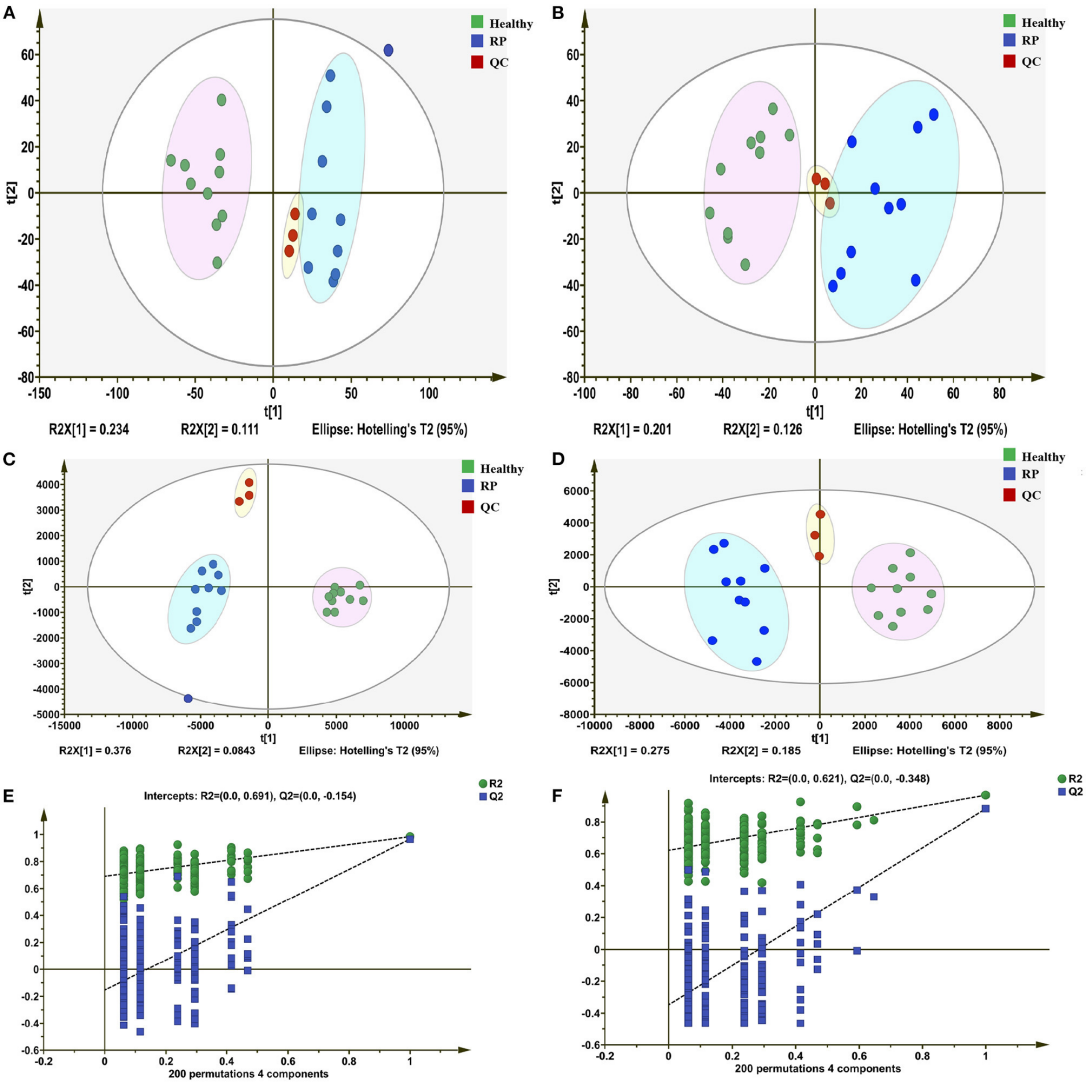
Differentiation of metabolic profiles of plasma of healthy dairy cows and dairy cows with retained placenta (RP). **(A,B)** Principal component analysis (PCA) score plots based on plasma metabolic profiles of healthy and diseased groups in positive and negative modes. ESI+: R^2^ = 0.568, ESI–: R^2^ = 0.603. **(C,D)** PLS-DA score plots of healthy and RP groups in positive and negative modes. ESI+: R2X = 0.235, R2Y = 0.953, Q2 = 0.451; ESI–: R2X = 0.257, R2Y = 0.969, Q2 = 0.684. **(E,F)** Permutation test of PLS-DA model: ESI+: intercepts of R^2^ = 0.691 and Q2 = −0.154, ESI–: intercepts of R^2^ = 0.621 and Q2 = −0.348.

In positive and negative ion modes, 4,617 and 2,897 metabolite ion peaks, respectively, were identified in positive and negative ion modes, and in these metabolite ion peaks, 3,012 metabolites were identified. In the generated PCA score plots, samples between groups showed a significant separation tendency, and samples within groups tended to cluster in positive and negative modes. The metabolic profiles of plasma samples from healthy and diseased groups were clearly separated in the negative and positive modes. These findings suggest that the plasma metabolic profile of dairy cows with RP was significantly different from that of healthy dairy cows (Figures 1A,B).

In the PLS-DA model, the samples of the disease and healthy groups were clearly separated (Figures 1C,D), and the Q2 regression lines based on a permutation test with a negative intercept suggested that the model was not overfitting (Figures 1E,F). In the positive and negative ionization modes, there were 629 and 488 metabolites, respectively, with VIP >1.

**Figure 2:**
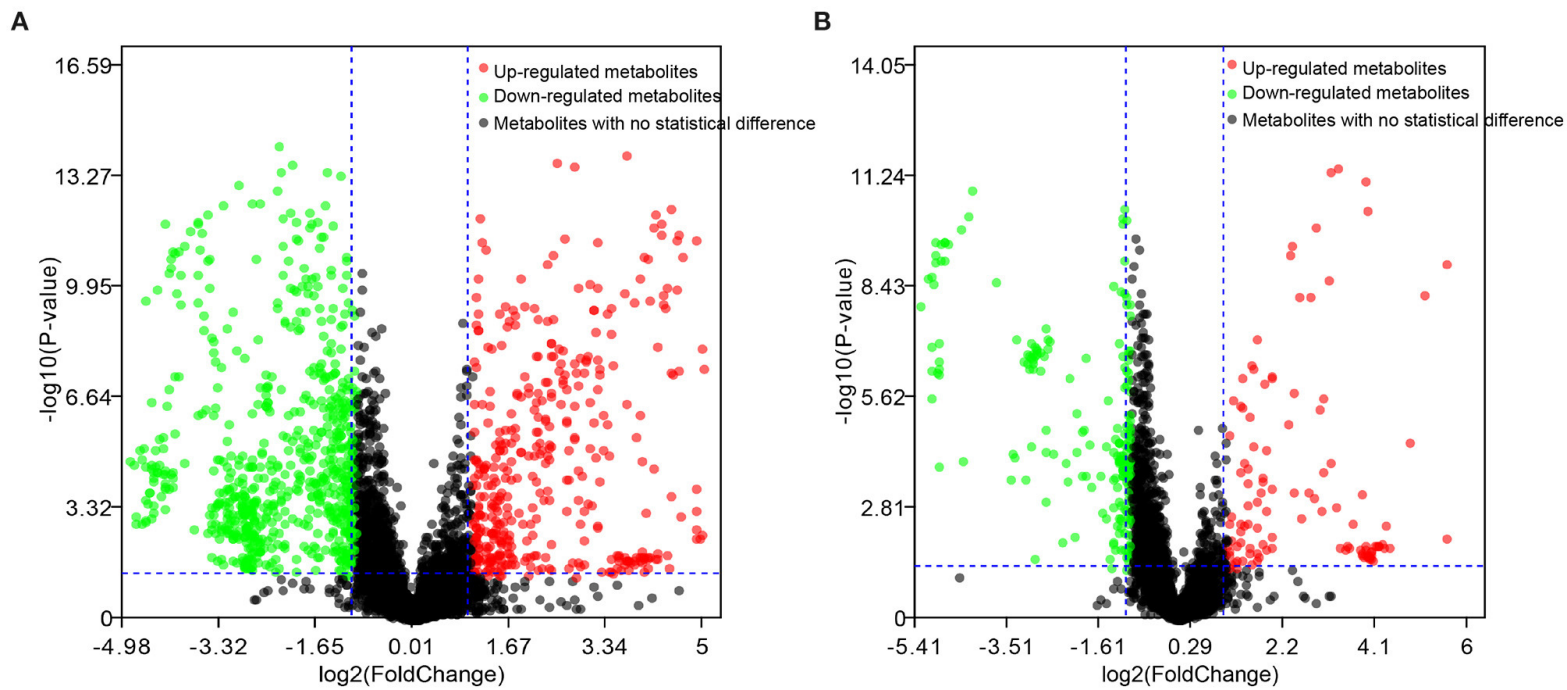
Candidate biomarkers in plasma of healthy dairy cows and dairy cows with RP. **(A)** Positive electrospray ionization (ESI+) mode; **(B)** negative electrospray ionization (ESI–) mode.

The differential metabolites in the plasma of dairy cows with RP and healthy cows were further screened, with an adjusted *p*-value < 0.05 and fold change >2. There were 164 and 112 differential metabolites with an adjusted *p*-value < 0.05 and fold change >2 in positive and negative ionization modes (Figure 2).

The differential metabolites were further optimized, with a VIP score >1, adjusted *p*-value < 0.05, and fold change >2.0 in positive and negative ionization modes to screen candidate biomarkers (Table 1). In the positive and negative ionization modes, 18 and 6 candidate biomarkers were found.

**Table 1:** Result of biomarkers identified in plasma of calves with RP.

**Metabolite**	**VIP**	**Adjusted *p*-value**	**Fold change (T/C)**	**SM**
l-Glutamate	2.3	0.000	12.3	+
Citrate	1.3	0.005	2.8	+
cis-Aconitate	1.2	0.003	3.0	+
Bilirubin	2.5	0.002	32.6	+
Phenylacetylglycine	1.1	0.002	2.5	+
l-Arginine	1.5	0.000	0.37	+
LysoPC (22:6)	1.2	0.000	0.45	+
LysoPC (22:5)	1.4	0.000	0.39	+
LysoPC (22:4)	1.4	0.000	0.37	+
LysoPC (20:4)	1.5	0.008	0.28	+
LysoPC (20:3)	1.3	0.000	0.39	+
LysoPC (20:2)	1.4	0.000	0.39	+
LysoPC (18:2)	1.4	0.000	0.39	+
LysoPC (16:0)	2.4	0.000	0.05	+
Deoxycholic acid 3-glucuronide	1.4	0.001	0.36	+
8,9-DiHETrE	2.0	0.000	8.39	+
Myristoleic acid	1.7	0.010	1.68	+
l-Lysine	1.1	0.000	0.54	+
l-Alanine	1.6	0.042	0.80	+
Salicyluric acid	1.3	0.000	0.484	–
Biliverdin	3.1	0.001	13.81	–
Leucine	1.6	0.002	0.49	–
LysoPC (18:3)	1.4	0.000	0.46	–

*RT, retention time; VIP, variable importance in projection; SM, scan mode; +, metabolites identified in positive electrospray ionization (ESI+) mode; –, metabolites identified in negative electrospray ionization (ESI–) mode. p < 0.05 compared with healthy dairy cows; T/C: dairy cows with RP compared with healthy dairy cows*.

**Figure 3:**
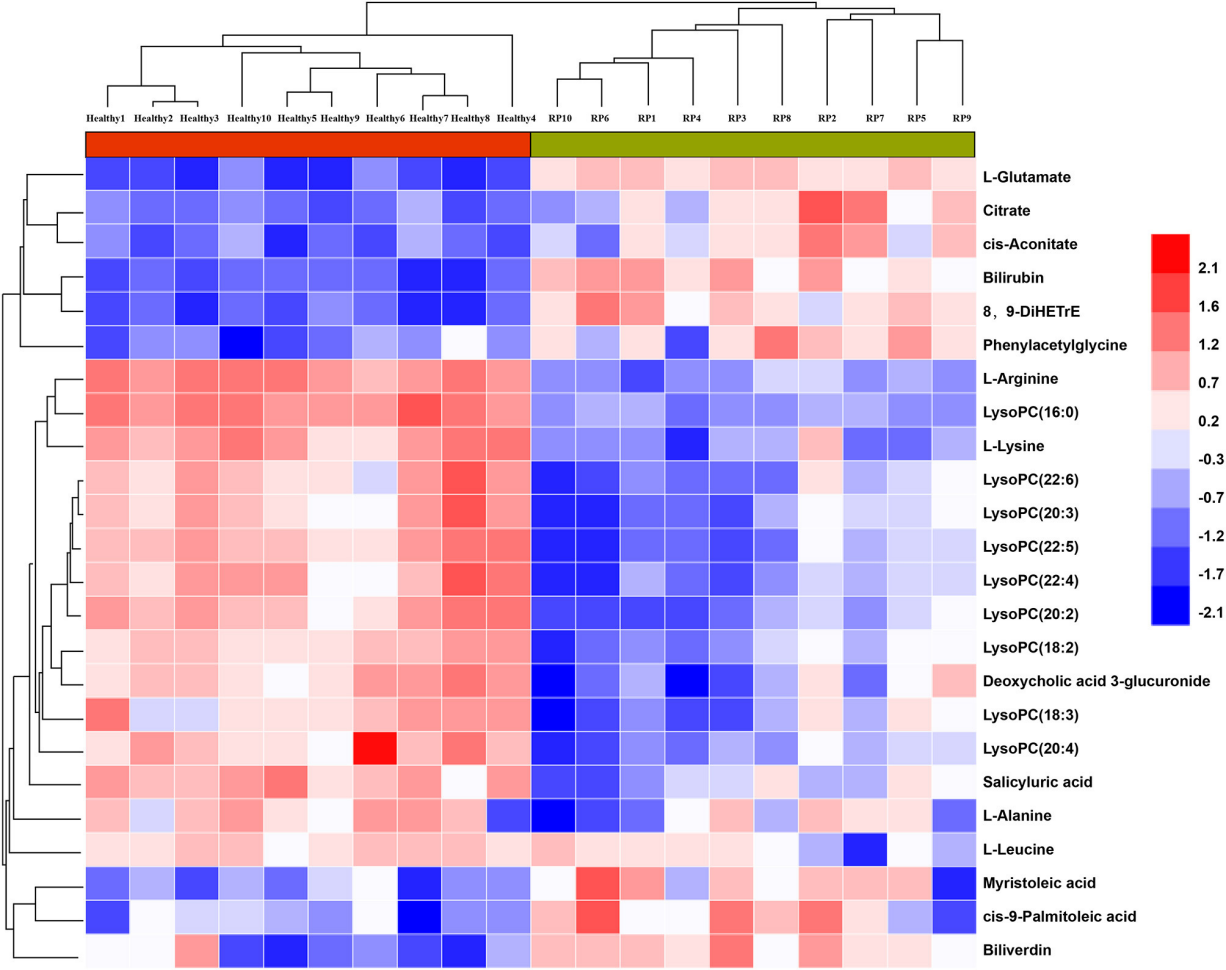
Relationship between healthy and RP samples, and expression patterns of potential biomarkers in different samples.

As shown in Figure 3, samples within groups formed clusters, and samples between groups were separated in positive and negative ionization modes. Candidate biomarkers with similar expression patterns in different samples were clustered, which suggested that these candidate biomarkers were located in a closer reaction process in the metabolic pathway.

**Figure 4:**
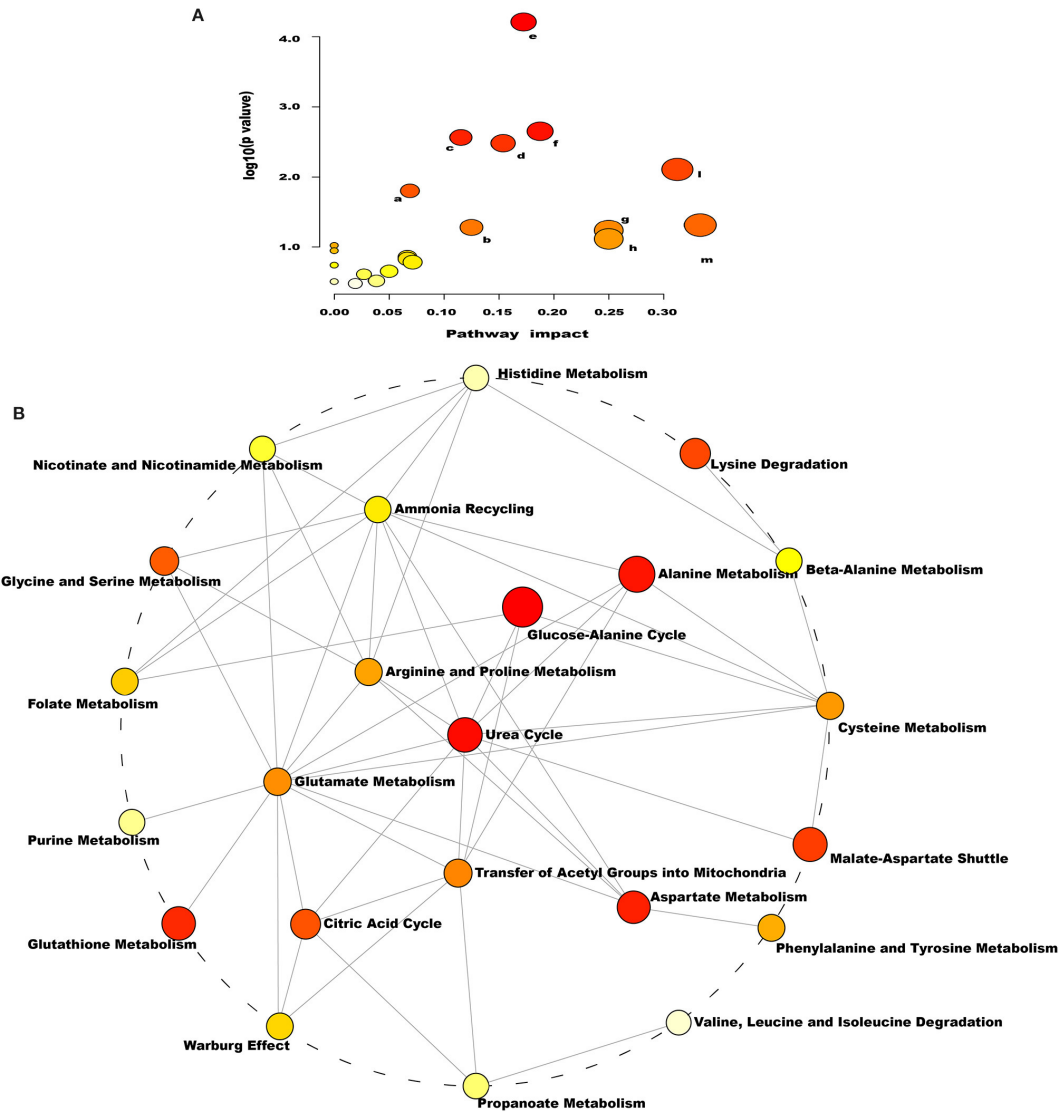
Pathways of conversion, utilization, and excretion of nitrogen were disturbed in dairy cows with RP. **(A)** Enriched KEGG pathway involving candidate biomarkers. a: citrate cycle; b: arginine and proline metabolism; c: porphyrin and chlorophyll metabolism; d: glyoxylate and dicarboxylate metabolism; e: aminoacyl-tRNA biosynthesis; f: alanine, aspartate, and glutamate metabolism; g: nitrogen metabolism; h: valine, leucine, and isoleucine; l: arginine biosynthesis; m: d-glutamine and d-glutamate metabolism. **(B)** Enrichment pathway network diagram involving candidate biomarkers. Each node represents a metabolite set, with its color based on fold enrichment.

As indicated by the enrichment analysis and pathway analysis shown in Figure 4, urea cycle, glucose–alanine cycle, ammonia recycling, arginine and proline metabolism, glutamate metabolism, and aspartate metabolism were significantly changed in dairy cows with RP. Moreover, these altered metabolic pathways were interconnected. These findings suggest that the conversion, utilization, and excretion of nitrogen were disturbed in these cows.

### Reduced Th1/Th2 Cytokine Ratio in Dairy Cows With RP

To probe the changes in the immune function of dairy cows with RP, the serum levels of IL-2, IL-4, IL-10, and TNF-α were detected. As shown in Table 2, the levels of IL-2 and IL-4 in serum of dairy cows with RP were significantly lower than those of healthy cows, while the levels of IL-10 and TNF-α increased, and the Th1/Th2 cytokine ratio was reduced.

**Table 2:** Reduced Th1/Th2 cytokine ratio, dysregulation of antioxidant capacity, and reproductive hormones in dairy cows with RP (mean ± SD).

**Indicator**	**Dairy cows with RP**	**Healthy dairy cows**
IL-2 (pg/L)	148.18 ± 3.09^*^	216.24 ± 11.45
TNF-α (pg/L)	222.66 ± 10.84^*^	315.60 ± 8.58
IL-4 (pg/L)	91.52 ± 1.30^*^	59.15 ± 1.75
IL-10 (pg/L)	60.33 ± 5.37^*^	44.45 ± 3.22
Th1/Th2	2.42 ± 0.07^*^	5.19 ± 0.23
GSH-Px (IU/ml)	60.85 ± 1.35^*^	79.12 ± 3.58
SOD (U/ml)	68.36 ± 1.81^*^	84.9 ± 2.12
MDA (nmol/ml)	6.00 ± 0.24^*^	3.66 ± 0.31
Progesterone (ng/ml)	6.25 ± 0.37^*^	2.89 ± 0.11
Estradiol (pg/ml)	21.43 ± 0.58^*^	29.19 ± 1.23
PGF2α (ng/ml)	1.91 ± 0.03^*^	3.15 ± 0.23

*IL, interleukin; TNF-α, tumor necrosis factor alpha; GSH-Px, glutathione peroxidase; SOD, superoxide dismutase; MDA, malondialdehyde*.

* *p < 0.05 compared with healthy dairy cows*.

### Imbalance of Antioxidant Capacity in Dairy Cows With RP

To investigate the alteration of antioxidant capacity in dairy cows with RP, the level of MDA and the activity of SOD and GSH-Px were detected. The results are shown in Table 2. The level of MDA significantly increased in the serum of dairy cows with RP compared with healthy cows. The activity of SOD and GSH-Px was significantly reduced in the serum of dairy cows with RP.

### Dysregulation of T-bil, ALP, and Reproductive Hormones

Alterations in blood biochemistry are important biomarkers in diseases. Therefore, blood biochemistry of dairy cows with RP was evaluated. RP was a common multifactorial postpartum reproductive disease, so reproductive hormones were also evaluated. As shown in Table 2 and Supplementary Table 3, increased levels of T-bil, ALP, and progesterone and decreased levels of estradiol and PGF2α were found in serum of dairy cows with RP.

## Discussion

To explore the complex pathogenesis of RP, increasing studies have focused on the detection of potential pathological factors involved in the complex pathological process of dairy cows with RP (30). Blood biochemical indicators are important markers of the physiological or pathological state of the body (31, 32). Therefore, in the present study, a few biochemical indicators were detected. The levels of T-bil and ALP significantly increased in dairy cows with RP, and other biochemical indicators (TP, ALB, GLB, ALT, AST, CK, BUN, CREA, GLU, TG, and TC) showed no differences between healthy and disease groups. ALP comes mainly from the liver and is also a marker of liver injury. However, because there were no differences in Alb, ALT, and AST, vital markers of liver injury, between healthy and disease groups, we speculated that the increased ALP in dairy cows with RP might be from the RP (33, 34).

The separation and expulsion of the placenta from the maternal uterus is a coordinated, regulated multi-system and multi-factor process. It has been demonstrated that the immune response plays a vital role in the process of separation and discharge of the placenta (16, 26, 27). During the perinatal period, Th1 cells are gradually derived from Th2 cells and secrete pro-inflammatory cytokines to initiate an inflammatory response, which induces apoptosis of trophoblast cells and endometrial epithelial cells and promotes placental separation (27, 35–37). In the present study, the Th1/Th2 cytokine ratio was significantly reduced in dairy cows with RP, which might decrease the inflammatory response in the uterus and cause the retention of placenta.

Moreover, it has been well-known that there is a close interaction between oxidation state and inflammation in the process of expelling fetal membranes (25, 26, 38). Oxidative stress increases the risk of placental retention (39). The result of increasing MDA and decreasing GSH-Px and SOD activity was consistent with a previous report (40). The balance of reproductive hormones is also essential for the separation and expulsion of the placenta (39, 41, 42). In this study, the levels of estradiol, progesterone, and PGF2α in serum of dairy cows with RP were significantly lower than those of healthy cows. Lower estradiol and PGF2α would reduce uterine contractility, causing failure to expel fetal membranes.

Although imbalanced antioxidant capacity, reduced Th1/Th2 cytokine ratio, and deregulation of T-bil, ALP, and reproductive hormones were uncovered, the interregulation of these biological factors and the exact pathogenesis of RP are still unclear. It is difficult to clarify the complex pathological process of RP involved in the nutritional metabolic, immune, nervous, and reproductive systems by using only a few blood indicators. Metabolites of the body are also products of the comprehensive regulation of multiple systems. Having an overview of changes in metabolites is beneficial to identify diagnostic markers and investigate pathogenic mechanisms of disease. Metabolomics can rapidly, sensitively, and comprehensively monitor alterations in the metabolites of the organism under physiological or pathological states (18, 20).

In the present study, plasma metabolomics were detected by ultra-high performance liquid chromatography–quadrupole time-of-flight mass spectrometry (UPLC-Q-TOF/MS) to reveal the potential biomarkers and pathogenesis of RP in dairy cows. The results suggest that metabolic profile significantly changes in dairy cows with RP. Moreover, 23 potential biomarkers were found and were mostly involved in urea cycle, glucose–alanine cycle, ammonia recycling, arginine and proline metabolism, glutamate metabolism, and aspartate metabolism. l-Arginine, l-lysine, l-leucine, and l-alanine, which were potential biomarkers, in serum of dairy cows with RP were significantly lower than those of healthy cows. Several previous studies had reported that there were significant differences in various amino acids, namely, leucine and arginine, between healthy dairy cows and dairy cows with RP, but in different research reports, these amino acids were no consistent trend of change (43–45). For instance, in several studies, significant decrease in various amino acids was detected by metabolomics in plasma of dairy cows with RP, and the corresponding results of the present study were consistent with it. However, in other studies, the corresponding amino acids appeared to have opposite results. Some researchers believe that the opposite results may be related to the different courses of the disease, but a unified view has not yet been formed, and further investigation is needed. It has been reported that arginine not only participates in the synthesis of protein, urea, and pyrimidine but also affects the release of various endocrine hormones, such as insulin and growth hormone (46, 47). Moreover, l-arginine may regulate immune function and estrogen by the NO pathway (48, 49). Lysine deficiency may also cause immunodeficiency (50). Decreased alanine in plasma is a sign of branched chain amino acid (BCAA) deficiency (51).

In this study, l-leucine, a type of BCAA, had a similar changing trend as l-alanine. It had been documented that BCAA deficiency could induce the dysfunction of immune cells (52). In this study, we speculated that decreased l-arginine, l-lysine, l-leucine, and l-alanine were responsible for imbalanced antioxidant capacity, reduced Th1/Th2 cytokine ratio, and deregulation of reproductive hormones. The lysophosphatidylcholines (LysoPCs), namely, LysoPC (16:0), LysoPC (22:6), LysoPC (20:3), LysoPC (20:5), LysoPC (20:4), LysoPC (20:2), LysoPC (18:2), LysoPC (18:3), and LysoPC (20:4), in serum of dairy cows with RP were significantly lower than those of healthy cows. Previous studies also showed lower concentrations of LysoPC (18:2), LysoPC (20:3), LysoPC (20:4), LysoPC (28:0), and LysoPC (28:1) in RP cows than in healthy cows (43, 45). Although LysoPC is a major component of lipids in blood, its physiological role is still unclear (53). LysoPC was reported to play a role in immunomodulation, anti-hemostasis, and cytotoxicity. Saturated LysoPC (LysoPC 16:0 and 18:0) and monounsaturated LysoPC (18:1) may induce inflammatory actions, such as release of chemotactic factors and enhanced production of reactive oxygen species (ROS) (53, 54).

We speculated that lower LysoPC reduced inflammatory response in the process of expelling placenta, which was consistent with the foregoing results that the Th1/Th2 cytokine ratio was significantly reduced in dairy cows with RP. Increased potential biomarkers, namely, l-glutamate, citrate, cis-aconitate, bilirubin, 8/9-DiHETrE, biliverdin, and phenylacetylglycine, were found in dairy cows with RP. Moreover, 8,9-DiHETrE is an important autocrine and paracrine factor that has diverse biological functions, such as regulation of vascular tone, renal tubular transport, and inflammation. Bilirubin and biliverdin have similar change trends as T-bil, suggesting that bilirubin metabolism is dysfunctional in dairy cows with RP. A previous study of metabolomics in dairy cows with RP had also showed that glutamate was significantly increased in different courses of RP (43). The increased l-glutamate, citrate, and cis-aconitate might be attributed to glutaminolysis. Glutaminolysis is composed of a series of biochemical reactions that catabolize glutamine to glutamate, citrate, aspartate, pyruvate, and lactate. In the present study, we speculated that glutaminolysis was significantly strengthened in dairy cows with RP. The result of KEGG pathway enrichment analysis based on these potential biomarkers suggested that the metabolic pathways involved are interconnected and the conversion, utilization, and excretion of nitrogen are disturbed in dairy cows with RP. Therefore, we considered that the regulation of metabolic pathways involved in these potential biomarkers could be a promising therapeutic strategy for RP and be also beneficial to elaborate the pathological mechanism of RP. However, since no samples were collected before the occurrence of RP, this study had certain limitations that whether these potential biomarkers could be used as potential early warning diagnostic biomarkers of dairy cows with RP still needed further study.

**Figure 5:**
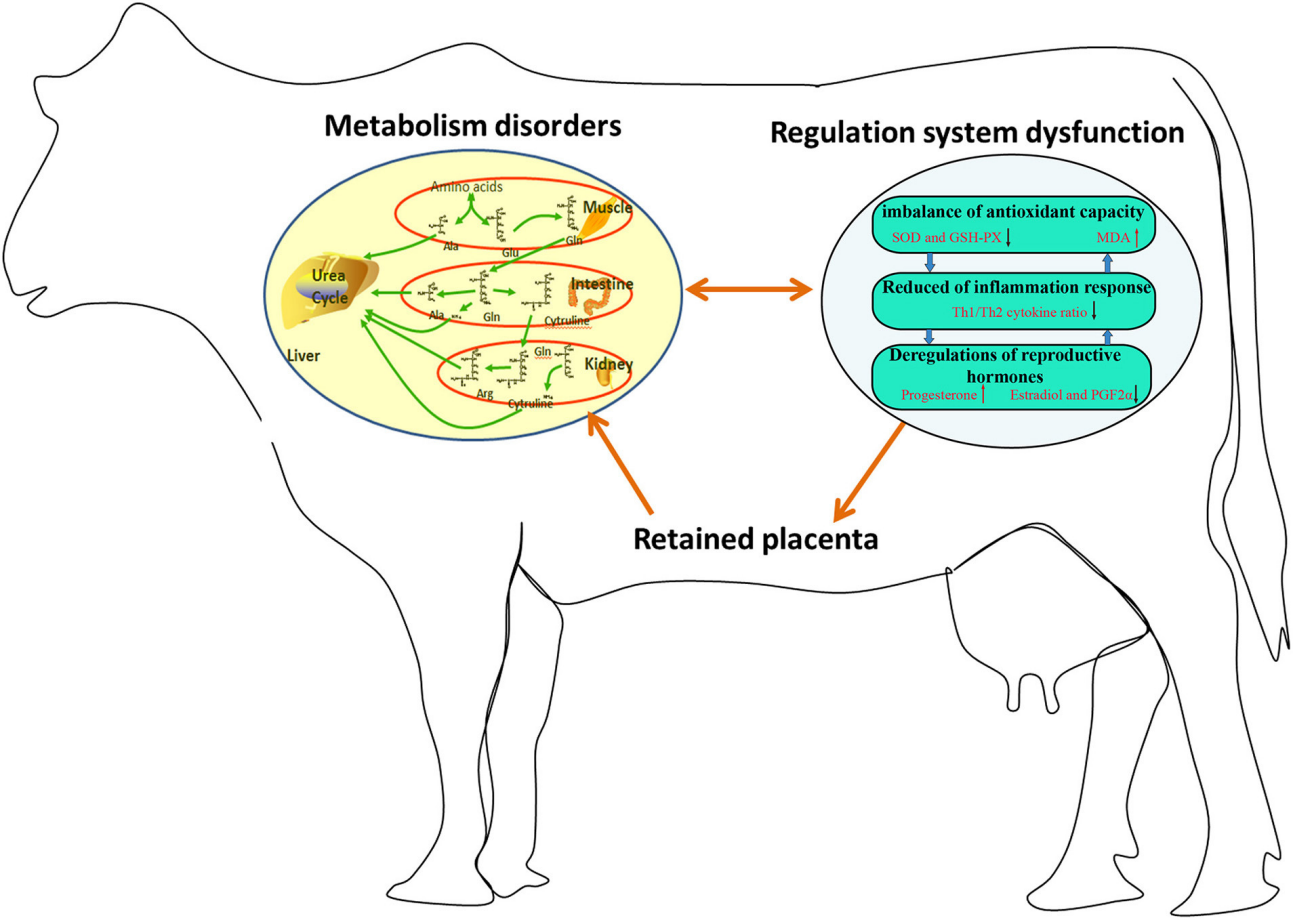
Imbalance of potential biomarkers might be responsible for imbalanced antioxidant capacity, reduced Th1/Th2 cytokine ratio, and deregulation of reproductive hormones in dairy cows with RP.

In summary, 23 potential biomarkers were uncovered in the plasma of dairy cows with RP. The metabolic pathways involved in these potential biomarkers were interconnected, and the conversion, utilization, and excretion of nitrogen were disturbed in these cows. Moreover, the imbalance of these potential biomarkers might be responsible for imbalanced antioxidant capacity, reduced Th1/Th2 cytokine ratio, and deregulation of reproductive hormones in these cows (Figure 5). The regulation of metabolic pathways involved in these potential biomarkers is a promising therapeutic strategy for RP.

## Data Availability Statement

The original contributions presented in the study are included in the article/Supplementary Material, further inquiries can be directed to the corresponding author/s.

## Ethics Statement

The animal study was reviewed and approved by the Animal Care and Use Committee of the Lanzhou Institute of Husbandry and Pharmaceutical Sciences of Chinese Academy of Agricultural Sciences (Lanzhou, China). Written informed consent was obtained from the owners for the participation of their animals in this study.

## Author Contributions

YL and DC: funding acquisition and methodology. ZZ: project administration and visualization. YY, XL, and LW: project administration. SW: resources. MH: conceptualization, methodology, formal analysis, and writing—original draft. All authors contributed to the article and approved the submitted version.

## Conflict of Interest

The authors declare that the research was conducted in the absence of any commercial or financial relationships that could be construed as a potential conflict of interest.

## Publisher's Note

All claims expressed in this article are solely those of the authors and do not necessarily represent those of their affiliated organizations, or those of the publisher, the editors and the reviewers. Any product that may be evaluated in this article, or claim that may be made by its manufacturer, is not guaranteed or endorsed by the publisher.

## References

[1] Benedictus L Koets AP Kuijpers FH Joosten I van Eldik P Heuven HC. Heritable and non-heritable genetic effects on retained placenta in Meuse-Rhine-Yssel cattle. Anim Reprod Sci. (2013) 137:1–7. 10.1016/j.anireprosci.2012.12.006 23317848

[2] Mahnani A Sadeghi-Sefidmazgi A Cabrera VE. Consequences and economics of metritis in Iranian Holstein dairy farms. J Dairy Sci. (2015) 98:6048–57. 10.3168/jds.2014-8862 26117350

[3] Moretti P Probo M Morandi N Trevisi E Ferrari A Minuti A. Early post-partum hematological changes in Holstein dairy cows with retained placenta. Anim Reprod Sci. (2015) 152:17–25. 10.1016/j.anireprosci.2014.11.019 25515153

[4] Mahnani A Sadeghi-Sefidmazgi A Ansari-Mahyari S Ghorbani GR Keshavarzi H. Farm and cow factors and their interactions on the incidence of retained placenta in holstein dairy cows. Theriogenology. (2021) 159:87–97. 10.1016/j.theriogenology.2020.10.007 33113449

[5] Zobel R Tkalčić S. Efficacy of ozone and other treatment modalities for retained placenta in dairy cows. Reprod Domest Anim. (2013) 48:121–5. 10.1111/j.1439-0531.2012.02041.x 22594457

[6] Qu Y Fadden AN Traber MG Bobe G. Potential risk indicators of retained placenta and other diseases in multiparous cows. J Dairy Sci. (2014) 97:4151–65. 10.3168/jds.2013-7154 24792789

[7] Dubuc J Denis-Robichaud J. A dairy herd-level study of postpartum diseases and their association with reproductive performance and culling. J Dairy Sci. (2017) 100:3068–78. 10.3168/jds.2016-12144 28161186

[8] Han IK Kim IH. Risk factors for retained placenta and the effect of retained placenta on the occurrence of postpartum diseases and subsequent reproductive performance in dairy cows. J Vet Sci. (2005) 6:53–9. 10.4142/jvs.2005.6.1.53 15785124

[9] Eiler H Hopkins FM. Successful treatment of retained placenta with umbilical cord injections of collagenase in cows. J Am Vet Med Assoc. (1993) 203:436–43. 8226224

[10] Stevens RD Dinsmore RP. Treatment of dairy cows at parturition with prostaglandin F2 alpha or oxytocin for prevention of retained fetal membranes. J Am Vet Med Assoc. (1997) 211:1280–4. 9373366

[11] Liu WB Chuang ST Shyu CL Chang CC Jack A Peh HC. Strategy for the treatment of puerperal metritis and improvement of reproductive efficiency in cows with retained placenta. Acta Vet Hung. (2011) 59:247–56. 10.1556/avet.2011.004 21665578

[12] Imhof S Luternauer M Hüsler J Steiner A Hirsbrunner G. Therapy of retained fetal membranes in cattle: comparison of two treatment protocols. Anim Reprod Sci. (2019) 206:11–6. 10.1016/j.anireprosci.2019.04.013 31103349

[13] Kimura K Goff JP Kehrli ME Jr Reinhardt TA. Decreased neutrophil function as a cause of retained placenta in dairy cattle. J Dairy Sci. (2002) 85:544–50. 10.3168/jds.S0022-0302(02)74107-6 11949858

[14] Dervishi E Zhang G Hailemariam D Dunn SM Ametaj BN. Occurrence of retained placenta is preceded by an inflammatory state and alterations of energy metabolism in transition dairy cows. J Anim Sci Biotechnol. (2016) 7:26. 10.1186/s40104-016-0085-9 27119014 PMC4845382

[15] Shimizu T Morino I Kitaoka R Miyamoto A Kawashima C Haneda S. Changes of leukocyte counts and expression of pro- and anti-inflammatory cytokines in peripheral leukocytes in periparturient dairy cows with retained fetal *membranes*. (2018) 89:1371–8. 10.1111/asj.13065 29956439

[16] Esposito G Irons PC Webb EC Chapwanya A. Interactions between negative energy balance, metabolic diseases, uterine health and immune response in transition dairy cows. Anim Reprod Sci. (2014) 144:60–71. 10.1016/j.anireprosci.2013.11.007 24378117

[17] Lu W Sun H Xu M Luo Y Jin J Shao H. Blood urea nitrogen may serve as a predictive indicator of retained placenta in dairy cows. Anim Reprod Sci. (2020) 218:106481. 10.1016/j.anireprosci.2020.106481 32507261

[18] Johnson CH Ivanisevic J Siuzdak G. Metabolomics: beyond biomarkers and towards mechanisms. Nat Rev Mol Cell Biol. (2016) 17:451–9. 10.1038/nrm.2016.25 26979502 PMC5729912

[19] Newgard CB. Metabolomics and metabolic diseases: where do we stand? Cell Metab. (2017) 25:43–56. 10.1016/j.cmet.2016.09.018 28094011 PMC5245686

[20] Wishart DS. Metabolomics for investigating physiological and pathophysiological processes. Physiol Rev. (2019) 99:1819–75. 10.1152/physrev.00035.2018 31434538

[21] de Seymour JV Conlon CA Sulek K Villas Boas SG McCowan LM Kenny LC. Early pregnancy metabolite profiling discovers a potential biomarker for the subsequent development of gestational diabetes mellitus. Acta Diabetol. (2014) 51:887–90. 10.1007/s00592-014-0626-7 25064235

[22] Pingili AK Kara M Khan NS Estes AM Lin Z Li W. 6beta-hydroxytestosterone, a cytochrome P450 1B1 metabolite of testosterone, contributes to angiotensin II-induced hypertension and its pathogenesis in male mice. Hypertension. (2015) 65:1279–87. 10.1161/HYPERTENSIONAHA.115.05396 25870196 PMC4433413

[23] Wang L Ko ER Gilchrist JJ Pittman KJ Rautanen A Pirinen M. Human genetic and metabolite variation reveals that methylthioadenosine is a prognostic biomarker and an inflammatory regulator in sepsis. Sci Adv. (2017) 3:e1602096. 10.1126/sciadv.1602096 28345042 PMC5342653

[24] Gonzalez-Dominguez R Sayago A Fernandez-Recamales A. High-throughput direct mass spectrometry-based metabolomics to characterize metabolite fingerprints associated with Alzheimer's disease pathogenesis. Metabolites. (2018) 8:52. 10.3390/metabo8030052 PMC6160963 30231538

[25] Endler M Saltvedt S Eweida M Åkerud H. Oxidative stress and inflammation in retained placenta: a pilot study of protein and gene expression of GPX1 and NFκB. BMC Pregnancy Childbirth. (2016) 16:384. 10.1186/s12884-016-1135-1 27923344 PMC5139037

[26] Yazlik MO Çolakoglu HE Pekcan M Kaya U Kaçar C. Vural MR, et al. The evaluation of superoxide dismutase activity, neutrophil function, and metabolic profile in cows with retained placenta. Theriogenology. (2019) 128:40–6. 10.1016/j.theriogenology.2019.01.020 30738254

[27] PrabhuDas M Bonney E Caron K Dey S Erlebacher A Fazleabas A. Immune mechanisms at the maternal-fetal interface: perspectives and challenges. Nat Immunol. (2015) 16:328–34. 10.1038/ni.3131 25789673 PMC5070970

[28] Edmonson AJ Lean IJ Weaver LD Farver T Webster G. A body condition scoring chart for holstein dairy cows. J Dairy Sci. (1989) 72:68–78. 10.3168/jds.S0022-0302(89)79081-0

[29] Pang Z Chong J Zhou G de Lima Morais DA Chang L Barrette M. MetaboAnalyst 5.0: narrowing the gap between raw spectra and functional insights. Nucleic Acids Res. (2021) 49:W388–96. 10.1093/nar/gkab382 34019663 PMC8265181

[30] Cruvinel LB Ayres H Zapa DMB Nicaretta JE Couto LFM Heller LM. Prevalence and risk factors for agents causing diarrhea (Coronavirus, Rotavirus, Cryptosporidium spp., Eimeria spp., and nematodes helminthes) according to age in dairy calves from Brazil. Trop Anim Health Prod. (2019) 52:777–91 10.1007/s11250-019-02069-9 31591674 PMC7089087

[31] Quiroz-Rocha GF LeBlanc S Duffield T Wood D Leslie KE Jacobs RM. Evaluation of prepartum serum cholesterol and fatty acids concentrations as predictors of postpartum retention of the placenta in dairy cows. J Am Vet Med Assoc. (2009) 234:790–3. 10.2460/javma.234.6.790 19284347

[32] Molefe K Mwanza M. Serum biochemistry in cows of different breeds presented with reproductive conditions. Onderstepoort J Vet Res. (2019) 86:e1–e7. 10.4102/ojvr.v86i1.1742 PMC6739554 31478736

[33] Gol M Sisman AR Guclu S Altunyurt S Onvural B Demir N. Fetal gender affects maternal serum total and placental alkaline phosphatase levels during pregnancy. Eur J Obstet Gynecol Reprod Biol. (2006) 128:253–6. 10.1016/j.ejogrb.2005.10.034 16332408

[34] Ferianec V Linhartová L. Extreme elevation of placental alkaline phosphatase as a marker of preterm delivery, placental insufficiency and low birth weight. Neuro Endocrinol Lett. (2011) 32:154–7. 21552196

[35] Norman JE Bollapragada S Yuan M Nelson SM. Inflammatory pathways in the mechanism of parturition. BMC Pregnancy Childbirth. (2007) 7 (Suppl. 1):S7. 10.1186/1471-2393-7-S1-S7 17570167 PMC1892064

[36] Boro P Kumaresan A Singh AK Gupta D Kumar S Manimaran A. Expression of short chain fatty acid receptors and pro-inflammatory cytokines in utero-placental tissues is altered in cows developing retention of fetal membranes. Placenta. (2014) 35:455–60. 10.1016/j.placenta.2014.04.009 24836458

[37] Boro P Kumaresan A Pathak R Patbandha TK Kumari S Yadav A. Alteration in peripheral blood concentration of certain pro-inflammatory cytokines in cows developing retention of fetal membranes. Anim Reprod Sci. (2015) 157:11–6. 10.1016/j.anireprosci.2015.02.011 25851495

[38] LeBlanc SJ Herdt TH Seymour WM Duffield TF Leslie KE. Peripartum serum vitamin E, retinol, and beta-carotene in dairy cattle and their associations with disease. J Dairy Sci. (2004) 87:609–19. 10.3168/jds.S0022-0302(04)73203-8 15202645

[39] McNaughton AP Murray RD. Structure and function of the bovine fetomaternal unit in relation to the causes of retained fetal membranes. Vet Rec. (2009) 165:615–22. 10.1136/vr.165.21.615 19933541

[40] Kankofer M. Antioxidative defence mechanisms against reactive oxygen species in bovine retained and not-retained placenta: activity of glutathione peroxidase, glutathione transferase, catalase and superoxide dismutase. Placenta. (2001) 22:466–72. 10.1053/plac.2001.0650 11373157

[41] Wischral A Nishiyama-Naruke A Curi R Barnabe RC. Plasma concentrations of estradiol 17beta and PGF2alpha metabolite and placental fatty acid composition and antioxidant enzyme activity in cows with and without retained fetal membranes. Prostaglandins Other Lipid Mediat. (2001) 65:117–24. 10.1016/S0090-6980(01)00123-X 11403498

[42] Wischral A Verreschi IT Lima SB Hayashi LF Barnabe RC. Pre-parturition profile of steroids and prostaglandin in cows with or without foetal membrane retention. Anim Reprod Sci. (2001) 67:181–8. 10.1016/S0378-4320(01)00119-1 11530264

[43] Hailemariam D Mandal R Saleem F Dunn S Wishart D Ametaj B. Metabolomics approach reveals altered plasma amino acid and sphingolipid profiles associated with patholological state in transition dairy cows. Curr Metabol. (2014) 2:184–95. 10.2174/2213235X03666141216201446

[44] Hailemariam D Mandal R Saleem F Dunn SM Wishart DS Ametaj BN. Identification of predictive biomarkers of disease state in transition dairy cows. J Dairy Sci. (2014) 97:2680–93. 10.3168/jds.2013-6803 24630653

[45] Dervishi E Zhang G Mandal R Wishart DS Ametaj BN. Targeted metabolomics: new insights into pathobiology of retained placenta in dairy cows and potential risk biomarkers. Animal. (2018) 12:1050–9. 10.1017/S1751731117002506 29032783

[46] Malaisse WJ Blachier F Mourtada A Camara J Albor A Valverde I. Stimulus-secretion coupling of arginine-induced insulin release. Metabolism of L-arginine and L-ornithine in pancreatic islets. Biochim Biophys Acta. (1989) 1013:133–43. 10.1016/0167-4889(89)90041-4 2669974

[47] Pedrinelli R Ebel M Catapano G Dell'Omo G Ducci M Del Chicca M. Pressor, renal and endocrine effects of L-arginine in essential hypertensives. Eur J Clin Pharmacol. (1995) 48:195–201. 10.1007/BF00198298 7589041

[48] Kang K Shu XL Zhong JX Yu TT. Effect of L-arginine on immune function: a meta-analysis. Asia Pac J Clin Nutr. (2014) 23:351–9. 10.6133/apjcn.2014.23.3.09 25164444

[49] Pekarova M Lojek A. The crucial role of l-arginine in macrophage activation: what you need to know about it. Life Sci. (2015) 137:44–8. 10.1016/j.lfs.2015.07.012 26188591

[50] Jankowski J Mikulski D Mikulska M Ognik K Całyniuk Z Mróz E. The effect of different dietary ratios of arginine, methionine, and lysine on the performance, carcass traits, and immune status of turkeys. Poult Sci. (2020) 99:1028–37. 10.1016/j.psj.2019.10.008 32036960 PMC7587641

[51] Palmer TN Caldecourt MA Snell K Sugden MC. Alanine and inter-organ relationships in branched-chain amino and 2-oxo acid metabolism. Rev Biosci Rep. (1985) 5:1015–33. 10.1007/BF01119623 3938302

[52] Nie CX He T Zhang WJ Zhang GL Ma X. Branched chain amino acids: beyond nutrition metabolism. Int J Mol Sci. (2018) 19:954. 10.3390/ijms19040954 PMC5979320 29570613

[53] Tan ST Ramesh T Toh XR Nguyen LN. Emerging roles of lysophospholipids in health and disease. Prog Lipid Res. (2020) 80:101068. 10.1016/j.plipres.2020.101068 33068601

[54] Plemel JR Michaels NJ Weishaupt N Caprariello AV Keough MB Rogers JA. Mechanisms of lysophosphatidylcholine-induced demyelination: a primary lipid disrupting myelinopathy. Glia. (2018) 66:327–47. 10.1002/glia.23245 29068088

