# Supplementary material for: Plasma Metabolomics Reveals Pathogenesis of Retained Placenta in Dairy Cows
Source: Front Vet Sci. 2021 Aug 11;8:697789. doi: 10.3389/fvets.2021.697789 (PMC8385782; doi:10.3389/fvets.2021.697789)
Supplement: Supplementary file 1 [file Table_1.DOC]

STable 1 Characteristics of Holstein dairy cows enrolled in the study.

| Group | Number | BCS | Parity | Age |
| --- | --- | --- | --- | --- |
| Retained placenta | 10 | 3.4 ± 0.6 | 2 | 3.4 ± 1.2 |
| Healthy | 10 | 3.3 ± 0.5 | 2 | 3.5 ± 1.1 |

BCS, body condition score.

STable 2 Optical UPLC gradient elution program of plasma samples

| Tim (min) | A% | B% | Column temperature | Flow rate (mL/min) |
| --- | --- | --- | --- | --- |
| 0 | 99.0 | 1.0 | 25 °C | 0.3 |
| 1.5 | 99.0 | 1.0 | 25 °C | 0.3 |
| 13 | 1.0 | 99.0 | 25 °C | 0.3 |
| 16.5 | 1.0 | 99.0 | 25 °C | 0.3 |
| 16.6 | 99.0 | 1.0 | 25 °C | 0.3 |
| 20 | 99.0 | 1.0 | 25 °C | 0.3 |

STable 3 Changes in biochemical indicators of dairy cows with RP (mean ± SD).

| Indicator | Dairy cows with RP | Healthy dairy cows |
| --- | --- | --- |
| CREA (μM) | 83.27 ± 15.88 | 85.38 ± 35.49 |
| AST (U/L) | 81.71 ± 22.69 | 82.95 ± 28.77 |
| ALT (U/L) | 18.08 ± 8.96 | 19.84 ± 6.19 |
| TP (g/L) | 52.73 ± 9.41 | 56.12 ± 11.7 |
| ALP (U/L) | 87.86 ± 37.77* | 62.33±33.61 |
| ALB (g/L) | 23.56± 3.88 | 24.05 ± 4.50 |
| GLB (g/L) | 29.18 ± 6.19 | 32.08±7.72 |
| T-bil (μM) | 8.81 ± 5.55* | 4.95 ± 1.57 |
| TC (mmol/L) | 1.70±0.55 | 1.962 ± 0.59 |
| TG (mmol/L) | 0.11 ± 0.03 | 0.09 ± 0.038 |
| CK (U/L) | 338.77 ± 205.38 | 310.94±152.54 |
| GLU (mmol/L) | 5.05 ± 0.79 | 5.05 ± 0.56 |
| BUN (mmol/L) | 4.47 ± 1.19 | 4.72 ± 1.12 |

CREA, creatinine; AST, aspartate aminotransferase; ALT, alanine aminotransferase; ALB, albumin; GLB, globulin; TC, total cholesterol; TG, triglycerides; CK, creatine kinase; GLU, glucose; BUN, urea. *P < 0.05 compared with healthy dairy cows.
